# Doctors and local media: a synergy for public health information? A controlled trial to evaluate the effects of a multifaceted campaign on antibiotic prescribing (protocol)

**DOI:** 10.1186/1471-2458-11-816

**Published:** 2011-10-19

**Affiliations:** 1 LOcal Campaign on Antibiotics ALliance (LOCAAL) study group

## Abstract

**Background:**

Use of information campaigns and educational interventions directed to citizens and supported by physicians, aimed at promoting the appropriate use of medicines, have been evaluated by several studies with conflicting results. These interventions are potentially relevant, favouring the reduction of unnecessary use of medicines and related risks. Several studies have specifically evaluated the promotion of the appropriate use of antibiotics in adults and children, with variable results. A controlled study is proposed to evaluate the feasibility and effectiveness of a multifaceted intervention aimed at reducing antibiotic prescription by increasing awareness on risks of their unnecessary use.

**Methods/design:**

Information will be provided to citizens through several media (posters, local TV, radio and newspapers, video terminals, websites of Local Health Authorities). Brochures with information on expected benefits and risks of antibiotics will be also available, either with direct access in waiting rooms and pharmacies or handed out and mediated by doctors. Physicians and pharmacists will get specific data on local antibiotic resistance. A small group of representative doctors have also actively participated in defining the campaign key messages. A sample of general practitioners and paediatricians will be trained in patient counselling strategies.

The information campaign will be implemented in two Provinces of Emilia-Romagna during the fall-winter season (November 2011-February 2012). Change in the overall prescribing rate of antibiotics (expressed as DDD per 1000 inhabitants/day) in the intervention area will be compared versus other areas in the same Region. Knowledge and attitudes of the general population will be evaluated through a phone and internet survey on a representative sample.

**Discussion:**

While the campaign messages will be mainly directed to the general population, doctors' prescribing will be assessed. The main rationale for this apparent discrepancy lies in the influence patients may have on physicians' prescribing behaviour (directly or indirectly) and in physicians' endorsement of the campaign goals, considering their participation in its design.

This study could observe a reduction lower than 5% in the prescribing rate of antibiotics. Such a reduction would be of public health relevance and would determine average savings of almost twice as much as the campaign costs.

## Background

Use of information campaigns and educational interventions directed to citizens and supported by physicians, aimed at promoting the appropriate use of medicines, have been evaluated by several studies with conflicting results. Evaluating the feasibility and effectiveness of these interventions is relevant, considering the influence citizens may have on physicians' prescribing habits, both directly (when patients request prescription filling) and indirectly (when physicians think their patients expect getting a prescription) [1]. Physicians could also be motivated in their prescribing behaviour by the implementation of information campaigns, especially if their active participation is requested [2]. Analysing population targets, campaign methods and available experiences can provide useful information for planning this kind of intervention, which may determine potentially relevant contributions to public health by reducing unnecessary use of medicines and related risks.

As for population targets: citizens are exposed to several information sources about the effects of medicines, either through traditional media (TV, journals, radio, etc) or through the internet with its constant evolution to more interactive tools (blogs and the many features of web 2.0). These information are often promoted by drug producers: in Europe, direct-to-consumer drug advertising is currently not allowed (although the European Commission is considering a worrisome proposal to relax this ban) [3] but citizens may be exposed to web contents and, above all, to information promoted by people and institutions sponsored by drug companies, also available through traditional media [4]. These circumstances can favour a biased view of benefits and risks of treatments. Information promoted by public health institutions has generally a weaker potential, since resources and planning are most of the times inadequate if compared with commercially oriented information. Adequate planning of public health initiatives and allocation of economic as well as human resources are necessary in order to provide citizens with an evidence-based, unbiased view of benefits and risks of drug treatments, and to favour their appropriate use.

Several studies have shown the potential of mass media on the use of health interventions [5]. Medicines represent a relatively fortunate topic for a public health information campaign: maybe, suggesting to use medicines wisely in view of their benefits and risks can influence behavioural change more than suggesting the adoption of healthy lifestyles, uneasy to practise in spite of their well known benefits.

Aside from the specific topic focused on, many other variables may influence the effectiveness of multifaceted educational campaigns. Defining one or more relevant target audiences and key messages are essential elements. A thorough evaluation of local contexts and a wise mix of information tools is necessary as well, taking lessons from those who do commercial information and advertising. ''Social marketing'' techniques may help the promotion of evidence-based knowledge. This term refers to the application of marketing techniques to the solution of social and health problems [6]. A social marketing approach to evidence-based information could take into account a planning process involving ''consumer'' oriented research, objective setting, identification of barriers and, consequently, of strategies and tools to facilitate the transfer of key messages

As for doctor's role: information campaigns could be more effective if physicians facilitate the transmission of information to their patients [7]. In order this potential to be unfolded, it may be necessary to work on doctors' motivations and skills in better conveying information to their patients, in spending some more time and energy to evaluate patients' expectations, possible obstacles to attitudinal and behavioural changes and eventually to find the right keys motivating them to such changes.

Practicing physicians can also provide substantial help in developing campaign messages, since they are in a relevant position to evaluate comprehensibility and potential effects of specific information in the context of patient expectations in primary care. Such participation can also increase their sense of ownership of the campaign goals and facilitate their endorsement [2], which can ultimately lead to increased attention to appropriate prescribing. Lastly, doctors may be influenced by the awareness that patients could be more informed about (and more sensitive to) the campaign topic [8].

Several studies have evaluated the effects of educational interventions focused on the appropriate use of antibiotics in adults and in children. A Cochrane systematic review, reporting the results of 39 among RCTs and observational studies [9], showed that such interventions can be successful especially when local context and barriers are adequately analyzed and addressed. Specifically, multifaceted interventions involving both physicians and patients/public, using written information material, educational meetings and mass media seem moderately effective, more than single interventions, in decreasing antibiotic prescribing for inappropriate indications (range of absolute differences: 3-33%); only one of these studies, carried out in Finland, showed a reduction in antibiotic resistance (erythromycin resistance was halted in four years). Another systematic review published in 2008, reporting the results of 43 among RCTs and observational studies, showed that educational strategies involving physicians (in particular active education through seminars and visits) and patient education strategies (through written material and mass media) are moderately effective in reducing unnecessary antibiotic use (median absolute difference: 10%) [10]. Several studies evaluating the impact of regional and national information campaigns on antibiotic use are available, carried out especially in Australia [11,12] and the United States [13-15], but also in Europe (for example in France [16]). In selected cases, a favourable cost effectiveness ratio was shown [13]. A recent survey under the auspices of the European Commission revealed that information campaigns may change people's behaviour in terms of antibiotic use: more than one out of 10 people remember to have received information on this issue and are willing not to take antibiotics without talking to a doctor [17]. Definitively, there seems to be room for improving citizens' knowledge about the correct use of antibiotics.

Within a wider project called ProBA project (progetto Bambini Antibiotici), in 2003 the Health Agency of the Emilia-Romagna Region carried out a survey on 633 paediatricians (453 working as family paediatricians and 180 working in hospitals) and 1388 parents, aimed at evaluating the main determinants of antibiotic prescriptions for children [18]. This survey showed that improving paediatricians' and parents' knowledge on benefits and risks of antibiotics, and suggesting that resistance is a problem at the community but also at the individual level, may favour their more appropriate use.

Starting from these considerations and from local and international experiences on educational campaigns, we propose a study to evaluate the feasibility and effectiveness of a multifaceted intervention on antibiotic prescription in the general population, aimed at reducing it by increasing awareness on risk of unnecessary use, thus reducing patients' requests for unnecessary prescription. The specific objectives are:

• primarily, to reduce the overall volume of antibiotic prescription;

• secondarily, to reduce total costs associated to antibiotic prescriptions; to evaluate knowledge, attitudes and reported behaviours of a sample of citizens on benefits and risks of antibiotic use; and to evaluate trends in antibiotic resistance (in a three year period after the intervention) from sputum, throat swab, urine and blood samples

We consider such intervention of high public health relevance, considering the limited development of new antibiotics and the public health threat due to antibiotic resistance, which is in part accounted for antibiotic use in humans. The proposed intervention will involve citizens, general practitioners and paediatricians in two Provinces of Emilia-Romagna. In addition to promoting the appropriate use of antibiotics, this project will help to evaluate the feasibility of carrying out information campaigns on benefits and risks of medicines promoted by Local Health Authorities, with the collaboration of local physicians.

## Methods/design

This study will be a community level, controlled, non randomized trial. An information campaign on benefits and risks of antibiotic use, in general and specifically in upper respiratory infections, will be implemented in the provinces of Modena and Parma, in the Emilia-Romagna region. Regional areas where no campaign will be implemented and no information provided to physicians either will be the control group; the presence of local policies on antibiotics that would possibly alter the comparison with intervention areas will be evaluated immediately before implementing the campaign, so that provinces which may supply misleading data would not be included in the control group. Anyway, prescription of antibiotics in the intervention and control areas shall be similar at baseline (no statistically significant differences).

Considering that the proposed educational intervention will be done at the community level and will be multifaceted using several media, and considering its implementation in only two provinces, we think a randomised design would not add strength to the study validity: in fact, it would be impossible to restrict exposure to half the population and there would be too few units of randomisation. Therefore, we choose a non randomised design which is more feasible given those circumstances.

### Study population

The proposed multifaceted intervention will target adult citizens and physicians. Specifically, citizens will be directly targeted by general information through brochures, posters and mass media. General practitioners and paediatricians will receive ad hoc information on antibiotic resistance and will have an active role in facilitating the campaign messages getting through, so that they may be also sensitized to the issue of appropriate antibiotic prescribing (Table 1).

**Table 1:** How the campaign is expected to affect the population targets

		POPULATION TARGETS	
	
		CITIZENS	PHYSICIANS
**LEVEL OF ACTION**	**KNOWLEDGE**	Awareness of the antibiotic resistance issue and of the importance of a correct use of antibiotics (through brochures, posters and mass-media)	Awareness of local data on antibiotic resistance (through a newsletter)
	
	**ATTITUDE**	Awareness that antibiotics are not a panacea, but their use is fundamental in specific circumstances	• Sensitization to the issue of appropriate antibiotic prescribing
			• Perception that patients may be more informed about (and more sensitive to) the antibiotic resistance issue
	
	**BEHAVIOUR**	• Lower pressure on doctors to get antibiotics	Appropriate antibiotic prescribing
		• Less antibiotic self-use	
		• More appropriate use	

### Intervention/exposure

The campaign will be generally focused on the appropriate use of antibiotics in upper respiratory tract infections. Problems arising from antibiotic overuse, in particular antibiotic resistance, will be particularly pointed at. On the other side, it will be highlighted that antibiotics are necessary in specific circumstances and should be used when doctors say it. The main instruments of the proposed multifaceted intervention are discussed as follows.

1. Written information material (brochures) targeted at the general population. Specifically, two different types of brochure will be available: type-A brochure will provide general information on benefits and risks of antibiotic therapies; type-B brochures will provide more specific information on three possible clinical situations patients (and doctors) may deal with: antibiotic is necessary and is prescribed; antibiotic is not necessary and is not prescribed; antibiotic may or may not be necessary, so that a "wait and see" period is warranted.

In developing the specific messages, particular attention has been given to the evaluation of key determinants of potentially inappropriate antibiotic use. The occurrence of antimicrobial resistance both at population and individual level, and of side effects, will be particularly targeted. In this regard, the relevance of antibiotic prescribing on individual resistance has been further highlighted by a recent systematic review and meta-analysis [19]. A small group of representative physicians (one per Health District, appointed by their Local Health Authorities) helped identifying the campaign key messages in order to maximize their relevance to the target audiences. Attention will be also given to the editorial appeal and clarity, borrowing communication techniques from commercial marketing to construct an attractive, concise, easy-to-read and recall format.

The brochures will be available in waiting rooms of general practices and local health services (type-A) and will be distributed to patients by general practitioners and paediatricians (type-B). Electronic versions will be available on the web. Translated versions in Albanian, Arab, Chinese, English, French, Polish, Rumanian, Russian will be also posted on the web, so that doctors could print them out to share information with their foreign patients (mostly immigrants).

2. Posters developed through the same methodology as described above (see point 1). Given the nature of this media, specific attention has been given to focusing on few clear messages. Posters will be mainly available in general practices, pharmacies and local health services.

3. Short videos to be transmitted through video terminals in highly accessed places (mainly pharmacies and waiting rooms in surgeries/health services), local TVs and websites of Local Health Authorities.

4. Radio-spots to be transmitted in local radio stations

5. Advertisements and concise articles in local newspapers, written in a scientifically sound but accessible way to the average reader.

6. Web materials/banners in web sites of local newspapers.

7. A newsletter on antibiotic resistance in Emilia-Romagna, available to physicians and pharmacists in the intervention areas.

8. Training courses in counselling strategies, directed to general practitioners and paediatricians to facilitate information transfer on the campaign contents. More specifically, all general practitioners and paediatricians in the intervention areas will get a half-day presentation about the campaign and its instruments, discussing the importance of counselling strategies through examples relevant to the antibiotics campaign. A six-module course will be pilot-tested on about 120-150 physicians on a voluntary basis during the campaign implementation, to improve their ability to convey information and facilitate their patients' behavioural change.

The proposed interventions will be implemented during a 4 month period (November 2011 - February 2012).

### Primary Outcome

Cumulative five-month (November to March) changes in prescribing rates of specific classes of antibiotics in general practice, expressed as DDD per 1000 inhabitants/day.

Data on antibiotic prescriptions (on beta-lactams, cephalosphorins, macrolides, fluoroquinolones, corresponding to J01C, J01D, J01F, J01M atc codes) will be retrieved from regional prescribing databases. They will include NHS prescriptions only from general practitioners (a small part of prescriptions may be made outside NHS on personal prescription books) and will not assess the actual use of antibiotics. In Emilia-Romagna, prescriptions from paediatricians are expressed in pieces and not in DDD, and will be evaluated separately.

### Secondary outcomes

- changes in antibiotic prescribing trends in eighteen months

- changes in antibiotic expenditure per 1000 inhabitants/day (comparing to the same November to March period from the previous year)

- knowledge of and attitudes about the campaign messages, through a telephone and internet survey on a sample of the target population.

As for trends in antibiotic resistance, the regional antibiotic surveillance system regularly monitors and will keep monitoring antibiotic resistance through the analysis of blood, throat swab and sputum samples. Considering this circumstance, data about antibiotic resistance will be evaluated in a three year period after the intervention.

### Sample size calculation

In the first three months of 2011, antibiotic prescribing rate in the provinces chosen as possible intervention areas was 21,1 DDD per 1000 inhabitants/day. Ratio of prescribing rates in intervention vs other Provinces in Emilia-Romagna was 1. Standard deviation of the distribution of antibiotic prescribed by general practitioners was about 3,0 in a 2007 dataset of antibiotic prescriptions in the province of Modena. Assuming that ratio of prescribing rates has not changed, that standard deviation of antibiotic prescription is 6,0 (a very conservative doubled figure compared to the 2007 sample) and that is similar in exposed and not exposed areas, it will be possible to observe tiny reductions of antibiotic prescription, and surely meaningful reductions of at least 3-5%, with alfa = 5% and beta = 10%. Potential average economic savings of such reductions would more than offset the fund received to finance the campaign and would be more than three times the out-of-pocket expenses to implement the campaign.

### Statistical analysis

T-test (for continuing variables), chi-square test (for discrete variables), time series analysis and multiple linear regression will be used, the latter to investigate in secondary analyses how specific variables (baseline prescriptions, province, health district, size of assisted population) can affect outcomes. Analysis of covariance (ANCOVA) will be used for evaluating primary outcomes in case relevant baseline imbalances will make statistical adjustment necessary.

### Ethics approval

The protocol has been approved by the provincial ethics committees of Modena and Parma

## Discussion

This study will explore the impact of a local multifaceted information campaign on antibiotics. While the campaign messages will be mainly directed to the general population, volume of antibiotics prescribed (therefore doctors' prescribing behaviour) will be the main outcome of interest. Three points make the rationale for this apparent discrepancy and for the relevant role that informing patients may play: prescribers may be influenced by actual or perceived patient pressure to get antibiotics; they may be influenced by the awareness that patients may be more informed about (and more sensitive to) the antibiotic resistance issue; they have been asked to actively participate in developing the campaign messages and facilitating their transmission to patients, so that their endorsement of the campaign goals may be more likely. Thus, the "complex" nature of the proposed intervention lies not only in the use of multiple instruments, but especially in such articulate interplay between targets of information and targets of behavioural change, mirroring some of the possible influences on drug prescription in clinical practice.

The potential impact of such a low-cost multifaceted intervention implemented within Local Health Authorities and using local media is of general interest, as well as its feasibility. One further reason of interest is that, in Italy, data are lacking about the impact of information campaigns addressing health topics, especially if "hard" outcomes (such as drug prescribing) are considered. The likely presence of a parallel national campaign implemented in the same period (as it was in 2009 and 2010, part of a European initiative) could theoretically weaken the potential impact of this intervention; on the other side, we would be more confident if any effect had to be shown and the added value of a local initiative, with the use of local media and especially the participation of physicians, would be further elucidated.

As for ethical aspects, the information campaign will provide general, clear and carefully balanced messages explaining when antibiotics are necessary and risks of their unnecessary use. This information is expected to increase appropriate use of antibiotics and, ultimately, health of the exposed population. Since doctors (in particular general practitioners) will have a fundamental role in mediating this information, the risk of its incorrect interpretation will be very limited.

## Competing interests

The author declares that they have no competing interests.

## Authors' contributions

This is a collaborative research, with a few research centres and professional groups involved (see the paragraph below). Each of the listed people within these centres have a relevant role in study implementation. Within the writing committee (see next paragraph) GF had the original idea and wrote the protocol while all other authors provided important intellectual contribution to study design and implementation. All the authors read and approved the final version.

## Authors' information

The study and the information campaign are coordinated by the proponent institution, CeVEAS (Centre for the Evaluation of the Effectiveness of Health Care), which has a key role in developing the information material, carrying out the training courses and evaluating prescribing and attitudinal data.

CeVEAS is a Department of the Local Health Authority of Modena, collaborating with several stakeholders such as Local Health Authorities and Regional Health Agencies, the Italian National Health Institute and the Italian Drug Evaluation Agency, and is a WHO Collaborating Centre for Evidence-Based Research Synthesis and Guideline Development in Reproductive Health. CeVEAS was founded in 1999 to evaluate health interventions and health policies (in particular pharmaceutical policies) mainly through the analysis of scientific literature, and to facilitate the access to the best evidence on health care through the transfer of relevant knowledge to physicians, policy makers and citizens. CeVEAS

The Emilia Romagna Regional Health Agency (Infectious Diseases Risk Unit) has a key role in analysing the determinants of antibiotic use, in evaluating topics and key messages of the information material as well as reviewing it, in evaluating antibiotic resistance data and in developing a newsletter on this topic.

The specific role of this Unit is to work on prevention and control of infectious diseases, with a special focus on healthcare associated infections, antimicrobial resistance, tuberculosis, and other emerging and re-emerging diseases (SARS, Chikungunya infections, West Nile disease).

The Unit activities consist of identifying and assessing interventions to implement within the regional health system: developing and implementing surveillance system for the diseases described above, monitoring of control programmes and implementation of organizational changes.

The Local Health Authorities of Modena and Parma, which are in charge of planning, organizing and providing health care services in the Province of Parma, will have a key role in organizing contacts with local media (journals, TV, video terminals licensees) and in organizing training courses for physicians. Representatives from these Units participate in developing a strategic plan for implementing the campaign.

General practitioners and paediatricians in the Provinces of Modena and Parma will have a key role in promoting the campaign messages, that they contributed to develop and evaluate

Pharmacies in the Provinces of Modena and Parma will have a key role in the dissemination of the information material and in providing information on the correct use of antibiotics

The communication agency CHANGE will have a key role in carrying out training courses in counselling strategies.

This agency, whose founders co-founded the Italian Society for Counselling in Medicine (SICIM), is a not for profit organization developing and disseminating throughout Italy a method of systemic counselling that can be applied to different professional contexts and is used, in particular, by health-care workers and professionals in helping relations with family systems in situations of difficulty, illness or distress.

## Pre-publication history

The pre-publication history for this paper can be accessed here:

http://www.biomedcentral.com/1471-2458/11/816/prepub
